# Introducing a gender-neutral pronoun in a natural gender language: the influence of time on attitudes and behavior

**DOI:** 10.3389/fpsyg.2015.00893

**Published:** 2015-07-01

**Authors:** Marie Gustafsson Sendén, Emma A. Bäck, Anna Lindqvist

**Affiliations:** ^1^Department of Psychology, Stockholm UniversityStockholm, Sweden; ^2^Department of Psychology, Gothenburg UniversityGothenburg, Sweden; ^3^Department of Psychology, Lund UniversityLund, Sweden

**Keywords:** gender-fair language, gender-neutral pronouns, attitude change, gender, hen

## Abstract

The implementation of gender fair language is often associated with negative reactions and hostile attacks on people who propose a change. This was also the case in Sweden in 2012 when a third gender-neutral pronoun *hen* was proposed as an addition to the already existing Swedish pronouns for *she (hon)* and *he (han)*. The pronoun *hen* can be used both generically, when gender is unknown or irrelevant, and as a transgender pronoun for people who categorize themselves outside the gender dichotomy. In this article we review the process from 2012 to 2015. No other language has so far added a third gender-neutral pronoun, existing parallel with two gendered pronouns, that actually have reached the broader population of language users. This makes the situation in Sweden unique. We present data on attitudes toward *hen* during the past 4 years and analyze how time is associated with the attitudes in the process of introducing *hen* to the Swedish language. In 2012 the majority of the Swedish population was negative to the word, but already in 2014 there was a significant shift to more positive attitudes. Time was one of the strongest predictors for attitudes also when other relevant factors were controlled for. The actual use of the word also increased, although to a lesser extent than the attitudes shifted. We conclude that new words challenging the binary gender system evoke hostile and negative reactions, but also that attitudes can normalize rather quickly. We see this finding very positive and hope it could motivate language amendments and initiatives for gender-fair language, although the first responses may be negative.

## Introduction

Language is seen as an important tool for determining gender, i.e., if something is being perceived as feminine or masculine (Boroditsky et al., 2003; Stahlberg et al., 2007), where gender most often imposes a dichotomy (Ansara and Hegarty, 2014). This implies that language also could be used as a tool for establishing gender-equality and to challenge gender perceptions. In Western culture and languages, actions toward gender-fair languages have primarily focused on making women more salient and reducing the so-called male bias (for a review, see: Stahlberg et al., 2007). For example, in the seventies, the feminist movement questioned the use of a generic masculine pronoun to refer to people in general (Moulton et al., 1978; MacKay, 1980; Phillips, 1981; Murdock and Forsyth, 1985).

The literature describes two types of gender fair language: “balancing/feminization’ and ‘neutralization.’ Feminization implies the use of gender-appropriate forms, and is more often used in languages with grammatical gender (e.g., German, French), for example by adding feminine versions to masculine titles (e.g., Lehrer/Lehrerinnen for masculine and feminine teachers; Stahlberg et al., 2001, 2007). Neutralization is more commonly employed in so called ‘natural gender languages’ (e.g., English, Swedish, Norwegian), and implies that gender-neutral forms are preferred over gendered forms. Examples are using the word *parents* instead of *mum* and *dad*, and *humankind* instead of *mankind* (at least in official records).

In Swedish, a recent action was to introduce the gender-neutral third person pronoun, *hen*, as a complement to the Swedish words for *she (hon)* and *he (han)* (Ledin and Lyngfelt, 2013; Milles, 2013; Bäck et al., 2015). In current time the word first appeared in 2012, figuring in a children’s book. In July 2014, it was announced that *hen* should be included in 2015th edition of The Swedish Academy Glossary (SAOL) constituting the (unofficial) norm of the Swedish language (Benaissa, 2014; Fahl, 2014), after what had been a long, sometimes offensive and heated debate in the media. No other language has so far added a third gender-neutral pronoun that actually has reached the broader population of language users, which makes the situation in Sweden unique. This article presents a review of the process on how *hen* became implemented, including the arguments that were put forward from opponents and proponents, respectively. We present data on attitudes toward *hen* during the recent 4 years and study how time is associated with the attitudes and actual use of the word.

The word *hen* is very similar to, and pronounced as, the Finnish gender-neutral pronoun *hän* with the same meaning, i.e., describing any person no matter their gender – although the language of Sweden’s cultural neighbor Finland belongs to the language group without gendered third-person pronouns (Stahlberg et al., 2007; Prewitt-Freilino et al., 2012). Even though the debate about *hen* took off in 2012, the word was first mentioned as early as in the 1960’s (Milles, 2013), when linguists proposed that a gender-neutral pronoun would be a more rational choice in comparison to a generic *he* or using double forms (i.e., *he and/or she*). However, these discussions were more of an academic nature limited to small linguistic communities and did not reach a broader public (Milles, 2013). In the beginning of the 21st century people in LGBT-communities (Lesbian-, Gay-, Bi-, Trans-) began to use *hen*, both for people outside the gender dichotomy and as a way of diminishing the salience of gender. A similar movement has been found in the English language, among linguists and among transgender communities, where more than 80 different forms of gender-neutral pronouns have been proposed. Today, one trend in English is to use gender-neutral pronouns such as *zie* and *hir* (Baron, 1986; Ansara and Hegarty, 2014; Love, 2014), although these words have not been very widespread outside the LGBT-communities (Crawford and Fox, 2007).

When the debate took off in 2012, the spark that started it was the publishing of a children’s book (Lundquist, 2012) that used *hen* to denote the main character of the book, instead of using a gendered pronoun. The author and the publisher also wrote a debate article in one of the largest newspapers in Sweden together with Karin Milles, a linguist researcher and advisor of gender-fair language planning, arguing for the introduction of a gender-neutral pronoun (Milles et al., 2012; Milles, 2013). Advocates of the word argued that children are too much influenced by gender categories, where non-gendered pronouns allow them to visualize and develop their stories much more freely (Milles et al., 2012). Antagonists argued that children listening to such non-gendered stories would be disoriented not knowing their gender, and that having a (binary) gender (i.e., being a girl or a boy) is something to be proud of (Lagerwall, 2012). At this point in 2012, the use of *hen* was highly controversial, which is illustrated by an incident when a columnist in one of the largest newspapers used *hen.* The reactions led the management of the paper to apply a policy against using *hen* in its news reporting (Cederskog, 2012). In contrast, an entertainment magazine changed all third personal pronouns into *hen* in their second issue in 2012 (Milles, 2013). Later in 2012, the Language Council of Sweden (*Språkrådet*) providing official recommendations about Swedish language, recommended that *hen* should not be used, since it could be irritating and conflict with the content in the text. This illustrates a common argument against gender-fair language reforms – where new forms are commonly described as awkward and potentially steeling attention from the message (Blaubergs, 1980; Parks and Roberton, 1998). For example the publication manual by American Psychological Association (APA) includes guidelines against sexist language stating that ‘…combination forms such as *he/she* or *(s)he* are awkward and distracting and are not ideal’ (APA, 2012, p. 74). APA recommends the use of ‘neutral’ words such as *the person*, or *they*. However, both *they* and *the person* might be associated with gender bias (most often a male bias), which existing literature on gender-fair language has shown is a robust phenomenon (e.g., Hyde, 1984; Stahlberg et al., 2001, 2007; Lenton et al., 2009; Garnham et al., 2012). According to the literature, a gender bias is described as the situation when care is taken to express gender-fairness in the language and people nevertheless seem to create biased perceptions where they associate the gender-neutral expressions with either a masculine or a feminine gender. For example in English, the word *they* could be used as an assumed generic form (Gastil, 1990; Strahan, 2008), but in a study where the generic *he* was replaced by *they*, children still more often associated *they* with a man (Hyde, 1984). Also, supposedly neutral words such as *person*, *mankind*, or even *human* have been associated with a male bias (Stahlberg and Sczesny, 2001; Douglas and Sutton, 2014; Bäck et al., 2015). These results imply that the creation of new words may be needed to override gender and cisgender bias, although it might take some time for language users to get used to them. However, the implementation of newly formed words is not an easy and straightforward enterprise, maybe especially not for a pronoun. From a linguist perspective, it has been argued that pronouns changes more slowly than other words because they belong to the so called ‘function words’ or ‘closed words classes’ (Milles, 2013; Paterson, 2014). Function words are used to organize the grammatical structure in a sentence and their meaning is only derived from how they are used in context (Chung and Pennebaker, 2007; Milles, 2013). Pronouns are organized in a grammatical system, thus adding a new word challenges the whole system (Paterson, 2014), which is not the case when nouns or verbs are added to a dictionary, or when feminine forms of professional roles are added to masculine forms.

*Hen* can be used in two different ways: either as a third-person pronoun in situations including general descriptions of an individual whose gender is unknown or is considered as irrelevant, or as a third-person pronoun in situations where the described person is not gender-neutral but describing someone identifying themselves outside the gender-dichotomy (Milles, 2013; Bäck et al., 2015). For people with a non-binary gender identity, double forms of pronouns (i.e., *he/she*) and guidelines for gender-fair language are excluding (Ansara and Hegarty, 2014). For example ‘APA’s binary descriptions of gender reinforce ethnocentric gender ideology that assumes ‘woman’ and ‘man’ are the only possible genders’ (Ansara and Hegarty, 2014, p. 264).

The different uses of *hen* align with the arguments from its proponents and antagonists. Representatives from LGBT-communities propose a gender-neutral pronoun since it dissolves gender expectations and includes all individuals no matter their gender-identity (Milles, 2013). These arguments have met the strongest reactions where the proponents have been targeted with offensive and hostile attacks. The antagonists have argued that queer people and feminists are trying to change biology, and that gender is one of the most natural categories. A maybe less controversial argument is that the gender-neutral pronoun *hen* is a shorter and more efficient way in comparison to double forms. Accordingly, *hen* could be used when gender is unknown, or as a generic pronoun. These arguments have been put forward by some feminists and linguists (Milles, 2013). Yet, other groups of feminists have been negative toward a gender-neutral pronoun since, they claim, it could be a way of diminishing women. For example, a well-known Swedish feminist and professor in literature has argued that the feminine gender is obscured by the word *hen* (Brattström, 2014). Hence, the use of *hen* and its consequences have not been agreed on, and disputes reside even within the feminist movement.

After 2012 followed a time with progress toward a more official implementation. In 2013, the Swedish Language Council *(språkrådet)* changed their recommendation and proposed that *hen* could be used as a gender-neutral pronoun, although with caution because it may distract attention from the message. The next year, in 2014, it was announced that the word should be included in the 2015th edition of the SAOL that constitutes the (unofficial) norm of the Swedish language (Fahl, 2014). In this year, the language council also formally changed their guidelines for gender fair language in public authorities, and included *hen* as an alternative to other neutral or gender balanced forms. Using *hen* is still not mandatory in official publications; each authority decides themselves whether to use it in public documents or not, and so far very few do (Ledin and Lyngfelt, 2013; Olsson, 2015).

In the Swedish media, the word has become more commonly used (Ledin and Lyngfelt, 2013; Milles, 2013). For example, during the first 6 months of 2012 *hen* was mostly seen in a vivid debate about the word itself, while during the second half of 2012, the word was actually used in texts unrelated to the debate about the word, that is, as a gender-neutral pronoun. In one of the bigger newspapers in Sweden the occurrences of *hen* increased over a year, from 1 in 2010, to 9 in 2011 and to 113 in 2012 (Ledin and Lyngfelt, 2013). This means that though *hen* still is rare, an increase is undisputable. The analyzed paper is among one of the conservative papers, thus it was presumed that occurrences in more progressive papers may be higher, however, a quantification of this hypothesis has not yet been done. In an effort to understand how the media used the word (generic or transgender), Ledin and Lyngfelt (2013) showed that 15% of the occurrences were related to transgender use, whereas 85% corresponded to a practice when gender was unknown, irrelevant or, as a generic form.

Since the pronoun is new, there is still limited research about how the word is perceived and what consequences it might have. A few studies have tested whether *hen* decreases a male- and cisgender bias. In one study (Wojahn, 2013), 150 participants read a story about a cellphone user, referring to the person either as *he, he/she, hen*, or *he or she*. Results showed that *hen* evoked the least male bias and also less cisgender bias. In a previous study, we have shown that a person described as *hen* was more often remembered as a person of unknown gender, whereas a person described by a neutral word is more often remembered as having a masculine gender (Bäck et al., 2015).

Gender-fair language is often implemented over several years. It commonly starts with activist movements who propose a change. Since people have a preference for status quo (Jost et al., 2004; Samuelson and Zeckhauser, 2005; Crandall et al., 2009), and a preservation of traditional gender arrangements (Jost et al., 2008), new linguistic gender word forms may be negatively reacted upon. Proponents of non-sexist language have been attacked, words have been defined as being linguistically wrong or awkward (Blaubergs, 1980; Parks and Roberton, 1998), and feminine occupational titles have been evaluated more negatively than their masculine traditional form (Formanowicz et al., 2013). However, familiarity and exposure breeds liking (Zajonc, 1968), thus the attitudes may change the longer gender-fair language has been used (Eidelman et al., 2009; Moreland and Topolinski, 2010). Whether such attitude change occurs also for gender-neutral pronouns within a country has not been studied before.

In studying the implementation process of gender-fair language reforms and the consequences on population attitudes and use, it is important to consider variables traditionally associated with negative attitudes toward gender fair language. If we are to make a claim that gender fair language reforms will be successful, an important task for the present research is to show that time in use is important to include when studying attitudes and frequency of use, together with other potential explanations. Previous research has identified a number of predictors of attitudes to gender-fair language and the following section will provide an overview of these.

*Sexism* in terms of attitudes toward gender equality has been identified as a predictor of negative attitudes toward gender-neutral language use (cf. Sarrasin et al., 2012), together with *political orientation* in terms of right-wing conservatism (Formanowicz et al., 2013; Norton and Herek, 2013). Also in the *‘hen-*debate,’ more left-wing than right-wing politicians used *hen* and promoted that *hen* should be included in the Swedish Dictionary (Milles, 2013). In Sweden, there are feminist movements both on the left and right of the political map, and in the last election more politicians than ever before openly stated that they considered themselves to be feminists (Öhberg and Wängnerud, 2014). Thus, feminist values would be associated with more positive attitudes, no matter of political orientation. Jacobson and Insko (1985) showed that feminist attitudes were associated with a higher use of gender fair language, such as using more double forms of pronouns. Feminist attitudes also mediated the effect between gender and attitudes toward gender fair language. Hence, even though the literature suggests that political right-orientation would predict negative attitudes, this is not entirely straight-forward, and we suggest that interest in gender issues may be a potent predictor as well.

*Gender* (as coded in a binary system feminine/masculine) as a predictor of attitudes to and use of gender-fair language has been inconclusive so far. Some studies have shown that women are more positive than men to gender-fair language (i.e., Prentice, 1994; Sarrasin et al., 2012) others have shown no differences (i.e., Koeser and Sczesny, 2014). Women tend to use gender-fair language more often than men (Koeser and Sczesny, 2014), and are more easily influenced to adjust to gender-fair language (Koeser et al., 2014). Notably, using a gender-neutral *hen* is not as clearly beneficial for women, as compared to other forms of gender-fair language (e.g., balancing masculine and feminine form, or avoiding masculine generics). Hence, it is not certain how, or even if, gender will affect attitudes to *hen*. Since *hen* challenges the binary gender system that is prevailing in most cultures, it could be argued that some individuals will show stronger resistance than others, depending on how important the gender system is to them. We argue that biological gender is not of greatest importance in this case, but rather the extent to which one identifies as a woman or a man, and how important this identification is. Indeed, arguments in the debate have touched upon such issues; for example, heterosexual people have argued that they are negative toward the word *hen* because it ‘restricts their right to express their gender identity,’ and that ‘romance between men and women will suffer’ (Lagerwall, 2012). Very few studies have investigated strength of gender identity as being a woman or a man in relation to gender-fair language. These studies have used forms of BEM Sex Role Inventory (BMSRI; Bem, 1974). The studies showed that a masculine gender identity (no matter of biological gender) was associated with more negative attitudes (Rubin and Greene, 1994), while androgynous gender identity has been associated with more positive attitudes, and higher use of gender-fair language (McMinn et al., 1990; Rubin and Greene, 1991). Given that Sweden is an egalitarian society, where the distinction between femininity and masculinity is no longer as strongly rooted in traditional feminine and masculine roles, we believe that the strength of *gender identity* is a better measure than gender roles as measured by BMSRI (Bem, 1974).

Finally, we believe that *age* will predict attitudes and the use, because younger people are more susceptible to new ideas and to challenge traditional roles, than older people are (Visser and Krosnick, 1998; Eaton et al., 2009).

The main purpose with the present research is to study how time and other factors are associated with change in attitudes and use of *hen*. In the present research we investigate the effect of time on the outcome variables. However, we do not here study the mechanism (for instance habituation) by which elapsed time can explain such effects, but rather show that other potential explanatory factors are not sufficient to explain the outcome effects alone. To date, time has been proposed as a cause for difference in evaluations (see for example, Sarrasin et al., 2012). However, no studies have followed an implementation over time in one language, with one specific word. It is also known from previous research that time has a positive effect on attitudes such that the longer something has been in effect the better people will like it (Zajonc, 1968; Moreland and Topolinski, 2010). We present data from 2012 to 2015, on the attitudes to *hen* and self-reported use of *hen* from 2013 to 2015. We make the following predictions:

H1. Attitudes towards *hen* will become more positive over time.
H2. Self-reported use of *hen* will increase over time.
H3. Sexism and right-wing political orientation will be associated with negative attitudes, as well a lower use of *hen*
H4. Age will be related to attitudes and use, such that younger people will be more positive, and indicate more use of the word, than older people. Gender is included as a control because some studies have shown that women are more positive to gender-fair language than men.
H5. A strong gender identity (as either a woman or a man) will be associated with more negative attitudes and less use. Interest in gender issues will be associated with more positive attitudes and higher use.
H6. Time will have a significant and independent effect on attitudes and use of *hen*, also when all other variables are controlled for.

## Materials and Methods

### Participants and Procedure

We have collected data on attitudes and use of *hen* at six points in time since 2012. Participants and the datasets are described in **Table 1**. Dataset 1 and 2 consist of participants that were approached in the waiting hall at the Central station in Stockholm. Dataset 3 and 4 consist of students at Lund University. Dataset 5 consists of participants that were approached in the city of Lund. Participation was rewarded with a lottery ticket in all these data collections. All studies from 2012 to 2014 were completed through ‘paper-and-pencil’ questionnaires. The experimenter distributing the questionnaire was present during the participation, but on a distance to provide confidentiality. Dataset 6 consists of participants recruited through advertisement on different Internet forums. 243 started to fill in the questionnaire, 190 completed it. Participation was not compensated. This study was carried out in accordance with Swedish national ethical standards put forth by the Central Ethical Review board and the Swedish Research Council and with written informed consent from all participants.

**Table 1:** Overview of the studies: time, sample size, participants mean age, gender distribution, and type of sample.

			Age	Gender	
Year	Dataset	*N*	*M* (SD)	Women/men (%)	Sample
2012	1	184	36.6 (18.8)	59/41	Community
2013	2	61	40.3 (17.3)	59/41	Community
2013	3	160	23.6 (6.6)	50/50	Student
2013	4	51	22.7 (3.7)	67/23	Student
2014	5	40	31.0 (12.7)	43/57	Community
2015	6	190	33.5 (9.7)	67/27^∗^	Community
Total		686	31.7 (14.2)	60/39	

*^∗^In 2015, 4% indicated a gender identity outside the binary system, and 2% did not indicate gender.*

### Variables

The attitude to *hen* was assessed with one item ‘What is your opinion about the gender-neutral pronoun *hen* in the Swedish language?’ (Responses were given on a 7-point response scale ranging from ‘1 = very positive’ to ‘7 = very negative’). A short text introduced to the question and explained that *hen* was a gender-neutral word that can be used as a complement to the Swedish words representing *she* and *he*.

Behavior *(use of hen*) was measured from 2013 and onward with one item ‘Do you use *hen* yourself?’ (Responses were given on a 7-point response scale ranging from ‘1 = No, never’ to ‘7 = Yes, always’).

From 2013, participants also indicated whether they previously were familiar with the word *hen* from before. Answers ranged on a 7-point scale from ‘1 = not al all’ to ‘7 = very much’. Because there were very small variations in the responses from 2013 to 2014, in 2015 we dichotomized this response option into ‘yes’ and ‘no.’

*Sexism* was measured with five items from the Swedish version of the Modern sexism scale (Ekehammar et al., 2000; e.g., ‘Discrimination against women is no longer a problem in Sweden’; ‘Humiliating treatments of women in adverts is unusual’; Answers in terms of agreement or disagreement were given on a 7-point scale from ‘1 = Strongly disagree’ to ‘7 = Strongly agree). Sexism was included in all six datasets. Means and SD over time are included in **Table 2**.

**Table 2:** Means and standard deviations for included variables for each year, respectively.

	2012 (*N* = 184)			2013 (*N* = 271)			2014 (*N* = 40)			2015 (*N* = 190)		
	*M*	SD	α	*M*	SD	α	*M*	SD	α	*M*	SD	α
Modern sexism	2.31_a_	0.64	0.75	2.59_b_	0.86	0.65	2.95_b_	1.11	0.78	2.11_a_	1.21	0.83
Political orientation	4.08_a_	1.85		4.08_a_	1.73		4.03_a_	1.53		3.94_a_	1.77	
Interest gender issues				4.40_a_	1.73		3.65_a_	1.89		5.11_b_	1.73	
Gender identity				4.87_a_	1.79	0.84	5.46_a_	1.60	0.96	4.38_b_	1.66	0.82

*Chronbach’s alpha is included for scale measures: modern sexism and gender identity.*

*Means with different subscripts are significantly different from each other.*

*Political orientation* was assessed with one item ‘On a political scale from left to right, where is your position?’ Answers were given on a 7-point scale from ‘1 = clearly to the left’ to ‘7 = clearly to the right’).

*Gender identity* was included from 2013 and onward. In 2013 and 2014 it was assessed with two items (e.g., ‘To be a woman/man is an important part of my identity,’ ‘To be a woman/man is important to me,’ measured on a 7-point scale from ‘1 = strongly disagree’ to ‘7 = strongly agree’). In 2015, we began measuring gender identity with a validated sub-scale from Luhtanen and Crocker (1992) collective identity scale (e.g., ‘My gender identity is an important reflection of who I am,’ ‘My gender identity is an important part of my self image,’ (measured on a 7-graded scale: ‘1 = strongly disagree’ to ‘7 = strongly agree’). We used the two positively framed items because these were most similar to the items we used in 2013 and 2014. The reason for this shift was to use a more well-established scale.

Interest in gender issues was indicated with one item ‘How interested are you in general in gender issues?’ The scale ranged from ‘1 = not at all’ to ‘7 = very much’. This variable was included from 2013 and onward.

*Age* and *gender* was given by participants in a free-text response in order to avoid cisgenderism (Ansara and Hegarty, 2014). These variables were included in all datasets.

In order to run the analyses, we collapsed all datasets into one. In the regressions, *Time* was included as a continuous variable for the years 2012–2015. This means that dataset 2–4 was collapsed into 1 year, 2013. Because there were different sample types we controlled for that factor.

## Results

### Attitudes to ‘hen’ and Changes Over Time

Virtually all participants responded that they were familiar with the word *hen*. In 2013 and 2014 more than 95% responded a 6 or 7 on the 7-point scale, while in 2015 99.5% responded ‘yes’ to the question if they were familiar with the word since before.

**FIGURE 1:**
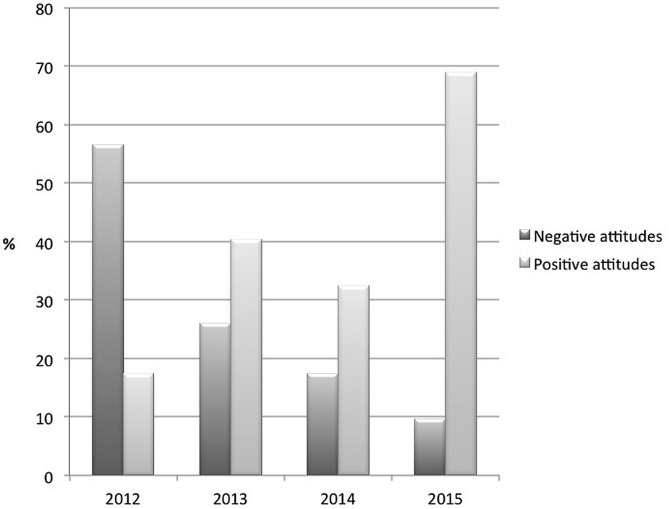
**Polarization of attitudes toward *hen* was reversed from 2012 to 2015.** ‘Negative attitudes’ = 1 and 2 on the rating scale; ‘positive attitudes’ = 6 and 7 on the rating scale.

The attitudes shifted from negative to positive over time (see **Table 3**). A univariate ANCOVA with year (2012, 2013, 2014, 2015) as the independent variable, sample type (student/community) as covariate, and attitude as the dependent variable, showed that attitudes changed over time, *F*(3,679) = 59.22, *p* < 0.001, = 0.21. *Post hoc* comparisons (Bonferroni adjusted for multiple comparisons), showed that the means did not change significantly from 2012 to 2013, or from 2013 to 2014, but between all other years (*p*’s < 0.004). Furthermore, the attitudes were polarized, such that respondents were either very negative or very positive toward the word *hen.* **Figure 1** shows that the very negative attitudes (i.e., 1 and 2 on the scale) decreased over time (2012 = 56.5%; 2013 = 26.1%; 2014 = 17.5%; 2015 = 9.6%); whereas the very positive attitudes increased (i.e., 6 or 7 on the scale; 2012 = 17.4%; 2013 = 40.4%; 2014 = 32.5%, 2015 = 68.9%).

**Table 3:** Means and SD for ‘attitude to *hen*’ and ‘behavior to use *hen*’ over 4 years (2012–2015).

	2012 (*N* = 184)		2013 (*N =* 271)		2014 (*N* = 40)		2015 (*N* = 190)	
	*M*	SD	*M*	SD	*M*	SD	*M*	SD
Attitude to *hen*	2.88_a_	2.17	4.38_a,b_	2.19	4.43_b_	2.02	5.71_c_	1.89
Behavior use *hen*			2.80_a_	1.98	2.80_a,b_	1.92	3.30_b_	1.47

*Significance was determined using Bonferroni test for multiple comparisons. Means for attitude and behavior with different subscripts are different from each other.*

### Use of ‘hen’ Over Time

**FIGURE 2:**
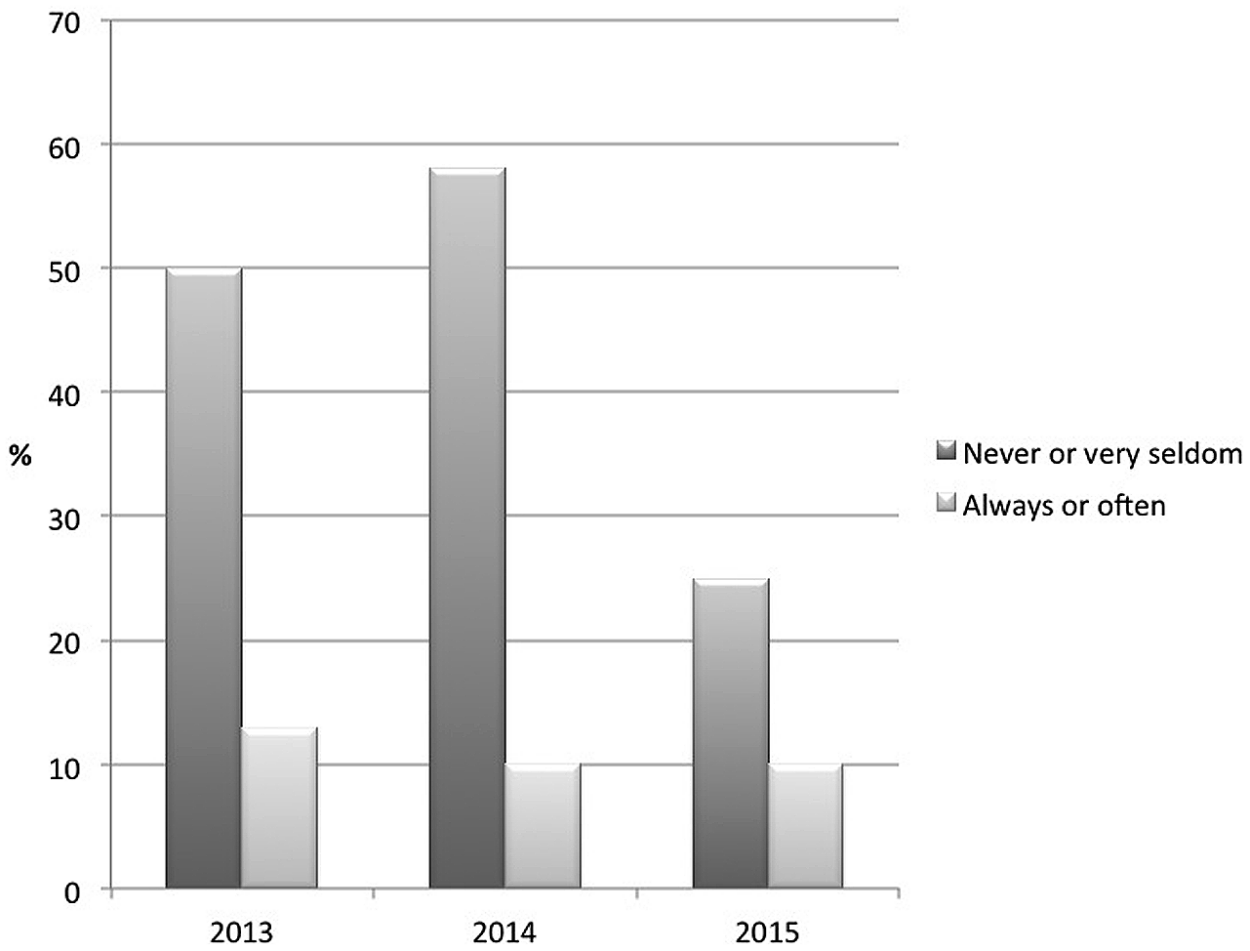
**Change in usage of a gender-neutral pronoun from 2013 to 2015.** ‘Never or very seldom’ = 1 and 2 on the rating scale; ‘Always, or often = 6 and 7 on the rating scale.

From 2013 and onward respondents also indicated whether or not they used the gender-neutral pronoun *hen* themselves (see **Table 3**). A univariate ANCOVA with year (2013, 2014, 2015) as independent variable, sample type as covariate, and behavior as dependent variable, showed a significant shift in behavior over time, *F*(2,498) = 8.56, *p* < 0.001, $\eta_{p}^{2}$ = 0.03. *Post hoc* pairwise comparisons (Bonferroni adjusted for multiple comparisons) showed that the difference was significant between 2013 and 2015, (*p* < 0.001). The responses for behavior were also somewhat polarized but not as much as for the attitudes, and were not reversed over the years (see **Figure 2**). A majority in 2013 (50%) and 2014 (58%) indicated they never or almost never used the word *hen* (as indicated with a 1 or 2 on the rating scale). In 2015, this group had decreased to 25%. However, there was no change in those who very often or always used the word *hen* (as indicated with a 6 or 7 on the rating scale) over time. In 2013, 13% responded they used *hen* often; in 2014 and 2015, 10% indicated they often used *hen.* Thus, both H1 and H2 stating that attitudes will become more positive and the use will increase over time were supported, although the attitudes changed more than the behavior.

### Predictors Associated with Attitudes and Use

Hypotheses 3 throughout 6 were related tttitude and use. To test the influence of time on the attitudes to *hen*, while also controlling for, and investigating effects of, the other predictors, a hierarchical regression with all the variables that were measured from 2012 to 2015 was computed. **Table 4** contains the correlations of included variables, collapsed across all years. The regression was performed in three steps (see **Table 5**). Regressions were also computed with dummy coding for time and the results were similar; we chose to present time variable as a continuous variable. The first step included time (2012–2014) and explained 19% of the variance. The longer the word has been known, the more positive were the attitudes. Adding sample type, age, and gender explained further 6% of the variance, such that being a woman, young, and a student was associated with more positive attitudes. Finally, in step 3, modern sexism and political orientation explained an additional 19% of the variance. Those with a right-wing orientation and higher sexism scores were more negative than individuals with left-wing orientation and lower sexism scores. When these factors were included, gender became insignificant, while time was still an important predictor. The total model explained 43% of the variance in the attitude to *hen.* Hence, H3, stating that sexism and political right affiliation would be associated with negative attitudes, H4, stating that younger people would be more positive to *hen*, and H6 stating that time will have an independent and significant effect on attitudes even when controlling for the other predictors, were all supported.

**Table 4:** Correlations, means, and SDs for variables included in regression.

	1	2	3	4	5	6	7	*M*	SD
(1) Attitude to *hen*	-							4.35	2.34
(2) Sample (0 = Community, 1 = Student)	0.075	-						1.31	0.46
(3) Age	-0.220**	-0.391*	-					31.67	14.28
(4) Gender (0 = Woman, 1 = Man)	-0.143**	0.057	0.001	-				1.40	0.49
(5) Modern sexism	-0.391**	0.153**	-0.074	0.274**	-			2.41	0.95
(6) Political orientation (high values = right-wing)	-0.374**	0.045	-0.063	0.076	0.448**	-		4.04	1.76
(7) Year	0.431**	-0.189**	-0.024	-0.093*	-0.096*	-0.03	-	2.32	1.14

^∗∗^Correlation is significant at the 0.01 level (2-tailed).*Correlation is significant at the 0.05 level (2-tailed). *N* = 672.

**Table 5:** Hierarchical multiple regression analyses predicting attitudes to *hen*.

	Step 1	Step 2	Step 3
	β	β	β
Time	0.435^∗∗∗^	0.440^∗∗∗^	0.424^∗∗∗^
Sample (0 = Community, 1 = Student)		0.097^∗^	0.132^∗∗∗^
Gender (0 = Woman, 1 = Man)		–0.115^∗∗∗^	–0.021
Age		–0.160^∗∗∗^	–0.184^∗∗∗^
Modern sexism			–0.270^∗∗∗^
Political orientation (high values = right wing)			–0.255^∗∗∗^

Δ*R*^2^	0.19^∗∗∗^	0.06^∗∗∗^	0.19^∗∗∗^
Total *R^2^*			0.43^∗∗∗^
*N*			647

*^∗^*p* < 0.05, ^∗∗^*p* < 0.01, ^∗∗∗^*p* < 0.001.*

From 2013 and onward three more variables were included in the questionnaires: *behavior (use of hen)*, *gender identity*, and *interest in gender issues*. In order to test whether gender identity and interest in gender issues account for more variance over sexism and political orientation, we calculated two hierarchical multiple regressions for attitude and behavior separately. The correlations, means, and SD are described in **Table 6**.

**Table 6:** Correlations, means, SDs for variables included in regressions.

	1	2	3	4	5	6	7	8	9	10	*M*	SD
(1) Attitude to *hen*	-										4.89	2.16
(2) Behavior, use *hen*	0.701**	-									2.97	1.81
(3) Sample (0 = Community, 1 = Student)	-0.113*	-0.003	-								1.43	0.50
(4) Age	-0.101*	-0.156**	-0.479**	-							29.82	11.66
(5) Gender (0 = Woman, 1 = Man)	-0.170**	-0.209**	0.084	0.028	-						1.39	0.49
(6) Modern sexism	-0.463**	-0.407**	0.149**	-0.069	0.283**	-					2.44	1.04
(7) Political orientation (high values = right wing)	-0.414**	-0.338**	0.064	0.006	0.083	0.469**	-				4.03	1.73
(8) Gender identity	-0.350**	-0.283**	0.028	0.022	0.019	0.213**	0.177**	-			4.74	1.75
(9) Interest gender issues	0.489**	0.460**	-0.087	0.058	-0.297**	-0.477**	-0.391**	-0.188**	-		4.60	1.79
(10) Year	0.292**	0.121**	-0.749**	0.257**	-0.133**	-0.212**	-0.036	-0.128**	0.176**	-	2.81	0.94

^∗∗^Correlation is significant at the 0.01 level (2-tailed). *Correlation is significant at the 0.05 level (2-tailed).

The regressions were computed in four steps to control for the contribution of variance in each step (see **Table 7**). For the *attitude* to the gender-neutral pronoun *hen*, time explained 9% of the variance in the first step, such that the longer *hen* had been in use, the more positive were the attitudes. The second step, where sample, gender and age were included, explained another 6% of the variance. Again, student samples were more positive than community samples, women were more positive than men, and younger people were more positive than older. The third step included sexism and political orientation, and explained another 21% of the variance, such that modern sexism and being right-wing oriented was associated with more negative attitudes. When these variables were included, gender became insignificant. The third step including gender identity and interest in gender issues, explained another 8%. Having a strong gender identity was associated with negative attitudes, whereas being interested in gender issues was associated with a positive attitude. When interest in gender issues and gender identity was introduced, neither gender or sample type were significant predictors, and the beta-weights for modern sexism and political orientation also decreased but remained significant. Although the beta-weight for time decreased in step 4 it remained significant.

**Table 7:** Hierarchical multiple regression predicting attitude to and use (behavior) of a gender-neutral pronoun *hen*.

	Attitude				Behavior			
	Step 1	Step 2	Step 3	Step 4	Step 1	Step 2	Step 3	Step 4
	β	β	β	β	β	β	β	β
Time	0.295^∗∗∗^	0.423^∗^	0.383^∗∗∗^	0.293^∗∗∗^	0.128^∗∗^	0.204^∗∗^	0.162^∗∗^	0.075
Sample (0 = Community, 1 = Student)		0.150^∗∗^	0.170^∗∗^	0.100		0.084	0.096	0.029
Gender (0 = Woman, 1 = Man)		–0.130^∗∗^	–0.028	0.010		–0.203^∗∗∗^	–0.111^∗∗^	–0.064
Age		–0.131^∗∗^	–0.129^∗∗^	–0.149^∗∗∗^		–0.158^∗∗^	–0.159^∗∗∗^	–0.180^∗∗∗^
Modern sexism			–0.282^∗∗∗^	–0.178^∗∗∗^			–0.270^∗∗∗^	–0.162^∗∗∗^
Political orientation (high values =right wing)			–0.273^∗∗∗^	–0.190^∗∗∗^			–0.203^∗∗∗^	–0.118^∗∗^
Interest gender issues				0.258^∗∗∗^				0.290^∗∗∗^
Gender identity				–0.186^∗∗∗^				–0.153^∗∗∗^
*N*				469				470
*ΔR*^2^	0.09^∗∗∗^	0.06^∗∗∗^	0.21^∗∗∗^	0.08^∗∗∗^	0.02^∗^	0.09^∗∗∗^	0.15^∗∗∗^	0.08^∗∗∗^
Total *R*^2^				0.44^∗∗∗^				0.32^∗∗∗^

*^∗^*p* < 0.05, ^∗∗^*p* < 0.01, ^∗∗∗^*p* < 0.001.*

In the regression with *behavior* (use of *hen)* as the dependent variable, time itself explained 2% of the variance. When age, gender, and sample type were included in the second step, those variables accounted for another 9% of the variance. Being older and having a masculine gender was associated with less use than being younger and having a feminine gender. The third step included modern sexism and political orientation and explained another 15% of the behavior. Right-wing orientation and sexism was associated with lower use of a gender-neutral pronoun. The fourth step, with gender identity and interest in gender issues explained another 9%, such that a strong gender identity was associated with lower use and being interested in gender issues was associated with a higher use. In the fourth step, time, gender, and sample type were no longer significant predictors. Also the beta-weights for political orientation and sexism decreased. The total model explained 32% of the variance. When controlling for all other factors, time contributed to a more positive attitude to a gender-neutral pronoun, although it did not increase the use of the pronoun *hen*. Thus, H6 was partially supported. Again hypothesis H3 and H4 were supported, and as predicted in H5, the strength of gender identity was a stronger predictor than gender itself. In addition, interest in gender issues proved to be a strong and independent predictor of both attitude and use. Even though it did not override the effect of political orientation, it should be taken as an indicator that this is an important aspect to take into consideration in future research.

Our results show that an introduction of a gender-neutral pronoun in the Swedish language was met with high resistance, but that both attitudes and behavior became more positive over time. We found that time predicted the attitude to *hen* also when other factors were controlled for. Other factors that contributed with unique variance to the attitude and the behavior were gender identity (but not gender itself), modern sexism, political orientation, and interest in gender issues.

## Discussion

This article has given an overview of the introduction of the new gender-neutral pronoun *hen* in the Swedish language. Data were collected during 4 years, starting in 2012 when the debate about a gender-neutral pronoun began and continued until 2015, 1 year after the word *hen* had been officially included in the Swedish dictionary.

### The Impact of Time

The results clearly show how the introduction of *hen* was associated with high resistance (in the media and among lay people), but also that attitudes became positive over time. In 2012, a majority of the study sample was explicitly very negative to the inclusion of a gender-neutral pronoun, whereas only a minority was very positive. However, already in 2013 this polarization was reversed, and in 2015 almost no one was very negative. A similar pattern was found for the use of the gender-neutral pronoun, although this change was smaller.

This is the first study about the introduction of gender-fair language analyzing the attitudes for a specific word over time. Previous research has proposed that variations in attitudes to gender-fair language could be due to how long it has been in use (see for example Sarrasin et al., 2012). This is the first study explicitly testing that hypothesis using data measurements at several time points. Indeed, time was the most important predictor of the attitudes, even after controlling for various other factors. This sends a very important message, because it should motivate language amendments also when there are strong reactions against an implementation.

We found that the attitudes changed faster than the behavior. The debate about *hen* was very wide-spread in the Swedish society, including the broader media landscape, leading to that the familiarity of *hen* very quickly included the large majority. Already in 2012, almost 95% of participants were familiar with the word, and in 2015, only 1 out of 190 participants were unfamiliar with *hen*. This may have been of importance for how fast the attitudes changed. For behavior to occur, *hen* must be activated and accessible in a specific moment (Fazio et al., 1989; Fazio and Olson, 2003; Glasman and Albarracín, 2006) as an alternative to, for example, double forms such as *she* or *he*. Because pronouns are often processed automatically (Chung and Pennebaker, 2007) the traditional system with *she* and *he* is probably still cognitively dominant over new forms of pronouns. Accessibility is although likely to increase over time, considering the increasingly widespread use of the word in media (Ledin and Lyngfelt, 2013), and in other arenas. For instance, the word was used in the lyrics of one of the songs to the Swedish contribution to the European Song Contest 2015, indicating its widespread acknowledgment. Social norms also facilitate behavior (Fazio, 1990), and it is plausible that people have been avoiding using *hen* because they still believe that the majority are negative to it. Thus, when people realize that the attitudes have changed, the word may be more common also among lay people and everyday users.

### Factors Explaining the Attitudes and Use

The more strongly participants identified themselves with their gender identity, the more negative attitudes they held and the least often they used the word. Women were somewhat more positive toward *hen* and used *hen* more often than men, but gender identity proved to be a much stronger predictor than biological gender. This supports the idea that a gender-neutral pronoun challenges the traditions of a binary gender system. These results also line up with previous research showing that androgynous gender roles were associated with a higher use of gender-fair language than traditional gender roles (Rubin and Greene, 1991). A large body of research indicates that people (especially adults) strongly prefer the system that they currently live in (Jost et al., 2004). People prefer to keep things stable and predictable. Any new word would thus probably elicit some resistance. However, there is reason to believe that a word explicitly challenging such a basic organizing principle such as the binary gender system elicits even stronger resistance. This resistance may also vary depending on individual factors. As was found in the present research a strong gender identity was negatively associated with attitudes toward *hen*, which can be considered a gender-fair amendment toward neutralization. However a strong gender identity might be positively related to amendments that add feminine alternatives to masculine forms because the binary gender-dichotomy would be even more strongly preserved and perpetuated with such amendments. This is an empirical question.

As in previous research, age, sexism, and political orientation was associated with attitudes to gender-fair language (Parks and Roberton, 2000; Sarrasin et al., 2012; Formanowicz et al., 2013). However, we also found that the influence of those factors decreased when gender identity and interest in gender issues were included. Even though political orientation still proved to be a significant predictor, this may indicate that interest is an important variable that eventually could diminish this effect, considering that there is a growing feminist movement also within the political right in Sweden.

Opinions associated with feminists may evoke higher resistance among people who do not actively endorse such values (Blaubergs, 1980). Thus, when the trendy entertainment magazine in one issue exchanged all third personal pronouns into *hen*, and when newspaper media started to use *hen*, this might have been of more importance than when feminists or linguists debated why *hen* should be used (Cialdini and Goldstein, 2004). Koeser et al. (2014) have also shown that the reading of gender-fair texts increases the use of gender-fair language. Hence, the fact that *hen* occurred more often in ordinary newspapers might have had a positive impact on use and might also imply an increase over the coming years.

*Hen* in Swedish was adapted from the gender-neutral Finnish word *hän* (Prewitt-Freilino et al., 2012). Maybe there are more words in gender-neutral languages that could be introduced either in natural or gender-marked languages. Some scholars have pointed to the need to be creative and come up with new words (Wayne, 2004), and borrowing them from other languages could be one strategy. New words might have a potential to override previous problems in applying gender fair language, since they may be less associated with a gender bias, which might be the case with other neutral words (Bäck et al., 2015). It should also be noted that both the Finish word *hän* as well as the Swedish gender-neutral pronoun *hen* very nicely fits into the Swedish system of pronouns, as being literally very close to, as well as alphabetically positioned between *han* (‘he’) and *hon* (‘she’). There is of course a risk that also *hen* could be associated with a male bias in future. Due to our results in this study we believe that such a risk is lower as long as *hen* is used as a generic or a transgender pronoun; however, this is an empirical question. When *hen* is broadly used in society, it is important to replicate studies that investigate how gender is activated when *hen* is used to refer to a person (Wojahn, 2013; Bäck et al., 2015).

### Limitations and Future Research

The design of the present study is cross-sectional and not longitudinal, which may imply selection bias in the samples and that other factors such as possible cohort effects may have had an impact on the results. With this in mind, we took care to collect both student and community samples, for which we controlled in the regression analyses. However, these samples were mainly drawn from cities and hence there may still be possible bias in the samples. This implies that generalization from the present study should be done with caution. With respect to cohort effects, our samples were fairly similar, although some minor deviations can be noted. The comparisons of the samples show that sexism was lower in the first year sample and the last year sample, gender interest was higher in the last sample, and gender identity was less strong in the last sample. Political orientation was similar in all samples. Here it can be noted that the last sample was collected using a web survey, which may imply selection bias since those who choose to participate can be expected to be relatively interested in issues of gender and language. With these problems in mind, we computed regressions for 2 years at a time, controlling for sexism, age, gender, and political orientation. Time was a significant factor in all three regressions (2012–2013; 2013–2014; 2014–2015).

This research is fairly explorative and the first of its kind. This entails that the items may not always have been entirely perfectly formulated. For instance, the response scale to the item ‘Do you use *hen* yourself?’ ranged from ‘No, never’ to ‘Yes, always.’ It may not be very clear to the participant what the response option ‘Yes, always’ entails, and this could be a contributing factor to why the results in general were weaker for the behavioral measure. ‘Always’ could indicate that one replace all personal pronouns with *hen*, or it could indicate that one always use *hen* when gender is unknown or irrelevant. Another limit is that this measure does not separate between written and spoken language. It is easier to use *hen* in writing than it is to use it in speaking. Future research should take these limitations into account when exploring how *hen* is used.

Language and communication have a large impact on the creation of a common ground and reality, for instance concerning what is considered as normal or desirable (Clark and Brennan, 1991; Hardin and Higgins, 1996). Thus, adding a gender-neutral pronoun to a natural gendered language may influence how individuals with a non-binary gender are perceived. In all our datasets ‘gender’ was an open-ended question, making it possible to self-categorize as neither woman or man. There were no such responses in 2012–2014, while in 2015, 4% (eight people) indicated a gender identity outside the gender dichotomy. Although it might be a coincidence, it could also be a consequence of the introduction of *hen*. This is something that could be further studied. A related important question that remains is what impact the use of *hen* actually has on representations of gender, and interpersonal attitudes.

We believe it is important to empirically test if common arguments proposed as negative consequences of gender-fair language are true. One such argument that remains to be tested is whether new word forms actually steel attention from the text content. If there is a cognitive load associated with *hen*, reading a text with *hen* should take longer time, and less information should also be recalled from such a text. Finally, it is important to note that we do not argue that time in use operates in isolation from other factors. One important aspect we believe is of great importance is that the Swedish society is becoming increasingly egalitarian and has a strong feminist movement, which includes people of all gender identities, and people with different political opinions. The fact that *hen* has its roots as far back as the 1960’s indicates that something else must have sparked the onset of the use in modern day than just time. One factor may be a societal ‘readiness’ to take this debate. Hence, societies of different levels of such readiness will of course receive a similar implementation differently. However, since there is a strong feminist movement in many societies, as indicated by the UN’s ‘heforshe’ campaign, we believe that the global readiness could be relatively favorable in a near future. Another factor is the word’s practical implications. In the Swedish case *hen* was introduced by LGBT communities and within the feminist movement, but clearly it met demands also among lay people as the word became as widespread as soon as it did.

## Conclusion

This is the first study analyzing the importance of time in implementing gender fair-language. The introduction of a gender-neutral pronoun in Sweden was firstly met with hostile reactions and negative attitudes, but over the course of only a couple of years, attitudes became largely positive. These results are positive for those working with gender equality and motivate implementations although the initial resistance may be high.

## Conflict of Interest Statement

The authors declare that the research was conducted in the absence of any commercial or financial relationships that could be construed as a potential conflict of interest.

## References

[B1] Ansara Y. G. Hegarty P. (2014). Methodologies of misgendering: recommendations for reducing cisgenderism in psychological research. *Fem. Psychol.* 24 259–270. 10.1177/0959353514526217

[B2] APA. (2012). *Publication Manual of the American Psychological Association* 6th Edn Washington, DC: American Psychological Association.

[B3] Bäck E. A. Lindqvist A. Gustafsson Senden M. (2015). Hen can do it: effects of using a gender neutral pronoun in a recruitment situation. *Paper presented at the The 8th Nordic Conferences on Language and Gender* Stockholm.

[B4] Baron D. (1986). *Grammar and Gender.* New Haven, CT: Yale University Press.

[B5] Bem S. L. (1974). The measurement of psychological androgyny. *J. Consult. Clin. Psychol.* 42 155–162. 10.1037/H0036215 4823550

[B6] Benaissa M. (2014). *Svenska Akademiens Ordlista Inför Hen [The Glossary of the Swedish Academy Includes Hen]*. Available at: http://sverigesradio.se/sida/artikel.aspx?programid$=$478&artikel$=$5924958 [accessed July 29, 2014].

[B7] Blaubergs M. S. (1980). An analysis of classic arguments against changing sexist language. *Womens Stud. Int. Q.* 3 135–147. 10.1016/s0148-0685(80)92071-0

[B8] Boroditsky L. Schmidt L. A. Phillips W. (2003). “Sex, syntax, and semantics,” in *Language in Mind: Advances in the Study of Language and Thought*, eds Getner D. Goldin-Meadow S. (Cambridge, MA: MIT Press) 61–79.

[B9] Brattström E. (2014). *Jag Tycker Det Skymmer Kvinnokönet [I Think it Obscures the Female Gender].* Available at: http://www.svd.se/kultur/saol-ingen-censurerande-instans_3784668.svd

[B10] Cederskog G. (2012). *Det Lilla Ordet Med Den Stora Laddningen [The Small Word With the Great Loading]*. Available at: http://www.dn.se/kultur-noje/det-lilla-ordet-med-den-stora-laddningen/

[B11] Chung C. K. Pennebaker J. W. (2007). “The psychological functions of function words,” in *Social Communication* ed. Fiedler K. (New York, NY: Psychology Press) 343–359.

[B12] Cialdini R. B. Goldstein N. J. (2004). Social influence: compliance and conformity. *Annu. Rev. Psychol.* 55 591–621. 10.1146/annurev.psych.55.090902.142015 14744228

[B13] Clark H. H. Brennan S. E. (1991). *Grounding in Communication.* Washington, DC: American Psychological Association 10.1037/10096-006

[B14] Crandall C. S. Eidelman S. Skitka L. J. Morgan G. S. (2009). Status quo framing increases support for torture. *Soc. Influ.* 4 1–10. 10.1080/15534510802124397

[B15] Crawford M. Fox A. (2007). IX. From sex to gender and back again: co-optation of a feminist language reform. *Fem. Psychol.* 17 481–486. 10.1177/0959353507084333

[B16] Douglas K. M. Sutton R. M. (2014). “A giant leap for mankind” but what about women? The role of system-justifying ideologies in predicting attitudes toward sexist language. *J. Lang. Soc. Psychol.* 33 667–680. 10.1177/0261927x14538638

[B17] Eaton A. A. Visser P. S. Krosnick J. A. Anand S. (2009). Social power and attitude strength over the life course. *Personal. Soc. Psychol. Bull.* 35 1646–1660. 10.1177/0146167209349114 19903975

[B18] Eidelman S. Crandall C. S. Pattershall J. (2009). The existence bias. *J. Pers. Soc. Psychol.* 97 765–775. 10.1037/a0017058 19857000

[B19] Ekehammar B. Akrami N. Araya T. (2000). Development and validation of Swedish classical and modern sexism scales. *Scand. J. Psychol.* 41 307–314. 10.1111/1467-9450.00203 11131952

[B20] Fahl H. (2014). *Hen Med i Ordlistan [Hen in the Dictionary]*. Available at: http://www.dn.se/kultur-noje/spraket/hen-med-i-ordlistan/ [accessed July 29, 2014].

[B21] Fazio R. H. (1990). Multiple processes by which attitudes guide behavior: the mode model as an integrative framework. *Advan. Exp. Soc. Psychol.* 23 75–109. 10.1016/S0065-2601(08)60318-4

[B22] Fazio R. H. Olson M. A. (2003). “Attitudes: foundations, functions, and consequences,” in *The Sage Handbook of Social Psychology* eds Hogg M. A. Cooper J. (London: Sage) 139–160.

[B23] Fazio R. H. Powell M. C. Williams C. J. (1989). The role of attitude accessibility in the attitude-to-behavior process. *J. Consum. Res.* 16 280–288. 10.1086/209214

[B24] Formanowicz M. Bedynska S. Cisłak A. Braun F. Sczesny S. (2013). Side effects of gender-fair language: how feminine job titles influence the evaluation of female applicants. *Euro. J. Soc. Psychol.* 43 62–71. 10.1002/ejsp.1924

[B25] Garnham A. Gabriel U. Sarrasin O. Gygax P. Oakhill J. (2012). Gender representation in different languages and grammatical marking on pronouns: when beauticians, musicians, and mechanics remain men. *Dis. Process.* 49 481–500. 10.1080/0163853X.2012.688184

[B26] Gastil J. (1990). Generic pronouns and sexist language: the oxymoronic character of masculine generics. *Sex Roles* 23 629–643. 10.1007/BF00289252

[B27] Glasman L. R. Albarracín D. (2006). Forming attitudes that predict future behavior: a meta-analysis of the attitude-behavior relation. *Psychol. Bull.* 132 778–822. 10.1037/0033-2909.132.5.778 16910754 PMC4815429

[B28] Hardin C. D. Higgins T. E. (1996). “Shared reality: how social verification makes the subjective objective,” in *Handbook of Motivation and Cognition* eds Sorrentino R. M. Higgins T. E. (New York, NY: Guilford Press) 28–84.

[B29] Hyde J. S. (1984). Childrens understanding of sexist language. *Dev. Psychol.* 20 697–706. 10.1037/0012-1649.20.4.697

[B30] Jacobson M. Insko W. Jr. (1985). Use of nonsexist pronouns as a function of one’s feminist orientation. *Sex Roles* 13 1–7. 10.1007/BF00287456

[B31] Jost J. T. Banaji M. B. Nosek B. A. (2004). A decade of system justification theory: accumulated evidence of conscious and unconscious bolstering of the status quo. *Polit. Psychol.* 25 881–919. 10.1037/0022-3514.85.5.823

[B32] Jost J. T. Nosek B. A. Gosling S. D. (2008). Ideology: its resurgence in social, personality, and political psychology. *Perspect. Psychol. Sci.* 3 126–136. 10.1111/j.1745-6916.2008.00070.x 26158879

[B33] Koeser S. Kuhn E. A. Sczesny S. (2014). Just reading? How gender-fair language triggers readers’ use of gender-fair forms. *J. Lang. Soc. Psychol.* 34 343–357. 10.1177/0261927x14561119

[B34] Koeser S. Sczesny S. (2014). Promoting gender-fair language: the impact of arguments on language use, attitudes, and cognitions. *J. Lang. Soc. Psychol.* 33 548–560. 10.1177/0261927x14541280

[B35] Lagerwall K. (2012). *Hen Gör Barnen Förvirrade [Hen makes children confused].* Available at: http://www.dn.se/nyheter/sverige/kritiker-hen-gor-barn-forvirrade/ [accessed February 14, 2012]

[B36] Ledin P. Lyngfelt B. (2013). Om bruket av hen i bloggar, tidningstexter och studentuppsatser [The use of hen in blogs, newspaper articles and student work]. *Språk Och Stil* 23 141–174.

[B37] Lenton A. Sedikides C. Bruder M. (2009). A latent semantic analysis of gender stereotype-consistency and narrowness in American English. *Sex Roles* 60 269–278. 10.1007/s11199-008-9534-z

[B38] Love A. (2014). A room of one’s own: afe placement for transgender youth in foster care. *N Y. Univ. Law Rev.* 89:2265.

[B39] Luhtanen R. Crocker J. (1992). A collective self-esteem scale: self-evaluation of one’s social identity. *Personal. Soc. Psychol. Bull.* 18 302–318. 10.1177/0146167292183006

[B40] Lundquist J. (2012). *Kiwi Och Monsterhunden [Kiwi and the Monster Dog]*. Stockholm: Olika Förlag.

[B41] MacKay D. G. (1980). Psychology, prescriptive grammar, and the pronoun problem. *Am. Psychol.* 35 444–449. 10.1037/0003-066x.35.5.444

[B42] McMinn M. R. Lindsay S. F. Hannum L. E. Troyer P. K. (1990). Does sexist language reflect personal characteristics? *Sex Roles* 23 389–396. 10.1007/BF00289227

[B43] Milles K. (2013). En öppning i en sluten ordklass? Det nya användandet av pronomenet hen [An opening in a closed word class? The new use of the pronoun hen]. *Språk Och Stil* 23 107–140.

[B44] Milles K. Salmson K. Tomicic M. (2012). *Det Behövs Ett Nytt ord i Det Svenska Språket [A New Word is Needed in the Swedish Language]*. Available at: http://www.svd.se/opinion/brannpunkt/det-behovs-ett-nytt-ord-i-svenska-spraket_6784859.svd

[B45] Moreland R. L. Topolinski S. (2010). The mere exposure phenomenon: a lingering melody by Robert Zajonc. *Emot. Rev.* 2 329–339. 10.1177/1754073910375479

[B46] Moulton J. Robinson G. M. Elias C. (1978). Psychology in action - sex bias in language use - neutral pronouns that arent. *Am. Psychol.* 33 1032–1036. 10.1037/0003-066X.33.11.1032

[B47] Murdock N. L. Forsyth D. R. (1985). Is gender-biased language sexist? A perceptual approach. *Psychol. Women Q.* 9 39–49. 10.1111/j.1471-6402.1985.tb00859.x

[B48] Norton A. T. Herek G. M. (2013). Heterosexual’s attitudes toward transgender people: findings from a national probability sample of U.S. adults. *Sex Roles* 68 738–753. 10.1007/s11199-011-0110-6

[B49] Öhberg P. Wängnerud L. (2014). Testing the impact of political generations: the class of 94 and pro-feminist ideas in the Swedish Riksdag. *Scand. Polit. Stud.* 37 61–81. 10.1111/1467-9477.12014

[B50] Olsson I. (2015). “Hen Och Andra Sätt Att Skriva Könsneutralt [Hen and Other Ways of Writing Gender-Neutral],” in *Klarspråak (A newsletter from the Swedish Language Council)*.

[B51] Parks J. B. Roberton M. A. (1998). Contemporary arguments against nonsexist language: blaubergs (1980) revisited. *Sex Roles* 39 445–461. 10.1023/A:1018827227128

[B52] Parks J. B. Roberton M. A. (2000). Development and validation of an instrument to measure attitudes toward sexist/nonsexist language. *Sex Roles* 42 415–438. 10.1023/A:1007002422225

[B53] Paterson L. (2014). *British Pronoun use, Prescription, and Processing* (Basingstoke: Palgrave Macmillan).

[B54] Phillips J. L. (1981). More on the pronoun problem. *Am. Psychol.* 36 694–694. 10.1037/0003-066x.36.6.694

[B55] Prentice D. A. (1994). Do language reforms change our way of thinking? *J. Lang. Soc. Psychol*. 13 3–19. 10.1177/0261927x94131001

[B56] Prewitt-Freilino J. L. Caswell T. A. Laakso E. K. (2012). The gendering of language: a comparison of gender equality in countries with gendered, natural gender, and genderless languages. *Sex Roles* 66 268–281. 10.1007/s11199-011-0083-5

[B57] Rubin D. L. Greene K. L. (1991). Effects of biological and psychological gender, age cohort, and interviewer gender on attitudes toward gender-Inclusive/exclusive language. *Sex Roles* 24 391–412. 10.1007/BF00289330

[B58] Rubin D. L. Greene K. (1994). Adopting gender-inclusive language reforms. *J. Lang. Soc. Psychol.* 13 91–114. 10.1177/0261927X94132001

[B59] Samuelson W. Zeckhauser R. (2005). *Status Auo Bias in Decision Making.* Northampton, MA: Edward Elgar Publishing.

[B60] Sarrasin O. Gabriel U. Gygax P. (2012). Sexism and attitudes toward gender-neutral language: the case of English, French, and German. *Swiss J. Psychol.* 71 113–124. 10.1024/1421-0185/a000078

[B61] Stahlberg D. Braun F. Irmen L. Sczesny S. (2007). “Representation of the sexes in language,” in *Social Communication* ed. Fiedler K. (New York, NY: Psychology Press) 163–187.

[B62] Stahlberg D. Sczesny S. (2001). Effects of the generic use of the masculine pronoun and alternative forms of speech on the cognitive visibility of women. *Psychol. Rundsch.* 52 131–140. 10.1026//0033-3042.52.3.131

[B63] Stahlberg D. Sczesny S. Braun F. (2001). Name your favorite musician - effects of masculine generics and of their alternatives in German. *J. Lang. Soc. Psychol.* 20 464–469. 10.1177/0261927x01020004004

[B64] Strahan T. E. (2008). ‘They’ in Australian English: non-gender-specific or specifically non-gendered? *Aus. J. Ling*. 28 17–29. 10.1080/07268600701877473

[B65] Visser P. S. Krosnick J. A. (1998). Development of attitude strength over the life cycle: surge and decline. *J. Pers. Soc. Psychol.* 75 1389–1410. 10.1007/BF00289330 9914661

[B66] Wayne L. D. (2004). Neutral pronouns: a moderst proposal whose time has come. *Can. Women Stud.* 24 85–92.

[B67] Wojahn D. (2013). De personliga pronomenens makt: en studie av hur pronomen styr våra föreställningar om personer [The power of the personal pronouns. A study on how pronouns direct person peception]. *Svenskans Beskrivning* 32 356–367.

[B68] Zajonc R. B. (1968). Attitudinal effects of mere exposure. *J. Personal. Soc. Psychol.* 9 1–27. 10.1037/h0025848

